# Leading multi-professional teams in the children’s workforce: an action research project

**DOI:** 10.5334/ijic.750

**Published:** 2012-01-13

**Authors:** Kaz Stuart

**Affiliations:** University of Cumbria and Brathay Trust, Ambleside, UK; Research Practice Leader (KTP), Brathay Trust, Clappersgate, Ambleside, Cumbria, LA22 0HP

**Keywords:** integrated, collaborative, children, action research

## Abstract

**Introduction:**

The 2004 Children Act in the UK saw the introduction of integrated working in children’s services. A raft of change followed with processes designed to make joint working easier, and models and theories to support the development of integrated work. This paper explores the links between key concepts and practice.

**Methods:**

A practitioner action research approach is taken using an autoethnographic account kept over six months. The research question was, to what extent is this group collaborating?

**Results:**

When the architecture of practice was revealed, differences between espoused and real practice could be seen. Whilst understanding and displaying the outward signs of an effective multi professional group, the individuals did not trust one another. This was exhibited by covert interprofessional issues. As a result, collaborative inertia was achieved. This realisation prompted them to participate in further developmental and participative action research.

**Conclusion:**

The paper concludes that trust and relational agency are central to effective leadership of multi professional teams.

## Introduction

The UK legislative framework directed professionals in the UK to work together for the benefit of children, young people and families. This way of working across previously divided services, such as education, health and social services was called ‘integration’. The process of working together involved collaborative practice. Policy mandated that a range of services integrated and ‘worked together’ across professional boundaries. This created complexity as professionals endeavoured to work together in new ways and there were practical and personal difficulties with the arrangements. Currently there are two million workers in the children’s workforce [1] trained in 60 separate professions that constitute the thirteen sectors of the ‘children’s workforce’ in the UK. They are organised into numerous integrated settings involving the public, private and third sectors. Some are located together geographically, others remain geographically separate but still work jointly, some share goals, and others pool budgets, so there are many contextualised forms of the deceptively simple term ‘integration’. The aim of integrated working was to ensure that no children fell through the gaps between services, and to reduce duplication of work by multiple services in a culture of increasingly high stakes accountability. This need had been highlighted by the failure of professionals to share information and cooperate in two high profile cases that led to the death of children (Victoria Climbie and Baby P). A number of tools were mandated that allegedly facilitated integrated working such as the ‘common assessment framework’, the role of the ‘lead professional’, a data base of information available to all services called ‘contact point’ and ‘information sharing’ protocols. Butthe education, health, social work and justice professionals that had to work together for the common good had their own professional backgrounds and discourses. They had their own terms and conditions and day-to-day practice based in their construct of ‘childhood’ and ‘youth’. The newly published ‘shared tools’ would not easily overcome these profound differences. Whilst there is some evidence of success [2–4] there were many professionals that found the process of integration difficult organisationally, professionally and personally. This research is situated in just such a group of professionals who found themselves in a newly organised interprofessional group focussing on workforce development in the children’s workforce in one local authority. I had been a member of this group for a year at which point I was offered a short-term contract to lead the group to more effective collaborative practice. I offered to document my experiences, both as a piece of PhD research for myself, and as a piece of action research for the group. The research question that we agreed on was “to what extent are we working collaboratively for the benefit of children and young people?”, and secondarily for me, “to what extent autoethnography could be used to analyse collaborative practice?”

## Theory

Before embarking on the leadership role, I decided to review a range of literature that might inform my thinking. This theoretical section therefore presents six key concepts that I took with me into the research situation, and that later became the conceptual and analytical framework.

In 2008 the Children’s Workforce Development Council (CWDC) announced that: *“Integrated working is where everyone supporting children, young people and families work together effectively to put the child at the centre, meet their needs and improve their lives”* [5].

The CWDC launched a tool to enable professionals to develop and self-assess their integrated working called the ‘One Children’s Workforce Tool’ [6]. This tool provided brief descriptions eight strands of integrated work located around a rainbow. There are two ends to each spectrum, one end is ‘integrated’ and the other ‘fragmented’. These are the descriptors taken from one of the strands:

Fragmented: “The individual agencies and personnel have different aims and ways of working, which means that there is not one common goal. The outcome is confused and children and young people aren’t helped as much as they could be.” [6].

Integrated: “New ways of working and new roles are encouraged, to make sure that everyone is working together. Professional expertise and skills are valued and used appropriately.” [6].

I suggest that these descriptions offer little tangible advice for leaders of services. There is no further detail. It remains the leaders task to apply these definitions, to assess their service and to decide what practical actions to take as a result I have selected the One Children’s Workforce Tool as one example of the over simplified and superficial support lent to professionals nationally. I now wish to introduce five concepts that reveal the complexity of the terrain and that can offer concrete forms of understanding and practical interventions for leaders.

**Figure 1.**
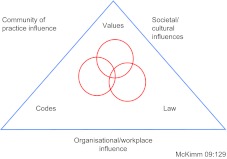
McKimm’s model of professional identities.

McKimm I suggest takes us closer to the heart of the difficulties in new integrated working arrangements. She [7] describes the importance of managing professional identities in integrated settings. She developed a model to help managers understand the complexity of an individual identity. Her model has a core of three overlapping circles within a triangle representing external context (Figure 1).

The core contains the values, attitudes and beliefs that an individual holds personally true and that are embodied in their profession. These are often encoded into the ethics and codes of practice, or even into the legal structures governing the profession. The external context exerts social and cultural influences on the professional to ‘act’ and ‘be’ as expected, through: stakeholder and client expectations, the engagement with a community of practice and colleagues in the workplace. Workplaces will also shape the identity of the individual through terms and conditions, procedures, ways of working and so on. So it can be seen that a police officer may have a very different way of intervening with young people than a youth worker due to differences in professional training (the areas inside the triangle). And, there may be variations in the practice of the police offers and the youth workers depending on the context that they are working in and the norms of the particular base that they are working from (the area outside the triangle). Establishing a shared goal may not therefore be as simple as the CWDC suggests above.

People in the children’s workforce with these different professional identities have to reach across multiple; departments, organisations and agencies, partnerships, networks, and sectors. This is called ‘boundary spanning’. As Peck and Dickinson state; “*boundary spanners are positions that link two or more systems whose goals and expectations are at least partially conflicting*” [8].

Working across organisational boundaries causes us to work with people with different professional identities and core values to ourselves. This can be the cause of consternation and conflict. Engström et al. [9], however, argue that professionals participation in multiple contexts and multiple communities of practice results in vertical models of expertise (socially learned or cognitively taught) that are too narrow, and propose a model of expertise that is socially constructed learning that creates expansive horizontal learning and expertise. People new to boundary spanning may feel out of their depth and to a degree unqualified and so learning: “*calls for the formation of new mediating concepts. In this sense, boundary crossing may be analysed as a process of collective cognitive formation*” [9]. Noble and Jones [10] research identified three types of distance between organisations, distance in autonomy, in culture and in caution (or risk). Arguably organisations that are well matched across each of these three areas would be easier to ‘span’. Organisations that have an outward focus to work, are aware of locality needs, are inclusive, invitational, empowering, share resources, have distributed leadership, and mobilise a range of people to take action are referred to as ‘bridged’. Bridged organisations contrast with those that are insular and ‘bonded’ [10]. Boundary spanning would also arguably be easier between organisations that are ‘bridged’ rather than ‘bonded’ [10]. This view is echoed by the National College of School Leadership [11], Anning et al. [12] and Easen et al. [13].

One aspect of collaborative practice that is intrinsic to working with other professionals across organisational boundaries is relationship. It is perhaps so intrinsic that it has become invisible, and it is absent from structural policy accounts of collaboration. Edwards [14] developed the notion of collaboration being relational work, in that:

*“Strong forms of agency may be required to help people who need to collaborate across organisational boundaries, to find moments of stability as they move in and out of different settings without the protection of institutional shelter”* [14].

Relational agency involved not only know how and know what, but also know who, Edwards suggested that it was no longer enough to work in isolation, professionals had to be networked and relational experts (i.e., able to strike rapport and build relationships in numerous different and varied organisations). Williams [15] agrees, stating that boundary spanners need strong interpersonal relationships, communication and political skills. Key to this is the ability to network. He also describes them as entrepreneurs and innovators, in that they are constantly navigating new ways of working. Hay group’s description of a professional that works in collaborative settings includes [16] the sense that they need to also be risk takers or rule benders as developing new ways of working cannot meet with the range of different organisational rules. Peck and Dickenson [17] stress the need for critical appreciation of the environment, understanding of different organisational contexts, prescience, and negotiation. Boundary crossers’, it is suggested would not have a typical career profile, but may develop knowledge of other organisations by a varied and diverse career profile [15], returning us to the horizontal dimension of expertise. There is some recognition in the literature [18–20] of the value of a relational approach to collaboration. Supporting Edwards notion of relational agency, Reder and Duncan [19] in their analysis of Child Abuse Cases have found that:

“the issues of communication are far more complex than has ever been envisaged by inquiry panels and that their more practical recommendations (especially those focussing on procedural and technical aides to improve message transfers) only address a small part of this complexity”.

What has become apparent in this limited review is that working across professions and across organisations is highly skilled and complex. This complexity was not apparent in the policy guidance on integrated practice which took a purely structural approach, perhaps as a structural approach seems simpler to implement than one that is relational. Simplifying something complex does not however mean that it will be easier to implement, and perhaps points to the use of simplification in rhetoric that seeks to convince leaders of the benefits of working together.

Arguably trust is fundamental to constructive relationships. As such, trust becomes a well-documented theme in the literature on boundary spanners’ attributes.

**Figure 2.**
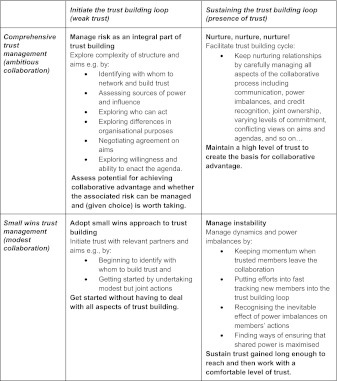
Huxham’s trust quadrants [23].

Trust is a contested term, but here it is enough to say that involves the hope that agreed expectations will be met. We trust on the basis of past experiences, reputation, shared characteristics and identity [21], yet 6, Leat, Seltzer and Stoker claim that there is a common practice of distrust in the business world. In collaboration, trust means the anticipation that people will do as expected [22]. Huxham [23] devise a four-way trust building quadrant that gives practical advice on how to build trust depending on the current level of trust (shown in Figure 2), and a trust cycle demonstrating the cyclical nature of positive trust exercises [23]. This grid acknowledges the complexity of a taken for granted attribute of collaboration (in its division into four situational quadrants) and offers practical tasks and steps for each.

Perri et al. [24] describe two contrapuntal drivers for trust: incentive driven or avoidance driven. This reframes an assumption that trust is a positive attribute. The NCSL [25] also position trust as critical to collaboration, placing it centrally in their diagram of collaborative working. The literature on trust thus helpfully conceptualises and illuminates ‘trust’ at a level not found in national guidance, arguably for reasons already cited.

When multi-professional teams work well together they achieve collaborative advantage. Huxham [23] describes leading collaborative endeavours as difficult. They are they say, full of complexity and ambiguity, and have dynamic memberships. In their model of collaborative leadership, they have four positive leadership activities—embracing, empowering, involving, and mobilising. They also identified two leadership activities that most leaders would not normally want to subscribe to; manipulation and politicking [23]. When these behaviours were enacted well then Huxham and Vangen [26] describe organisations as able to achieve collaborative advantage. They define this as the achievement of more than could be achieved by the partners alone. The opposite state is collaborative inertia where less was achieved than would be possible alone. These perhaps give the clarity that was needed in the One Children’s Workforce terms ‘fragmented’ and ‘integrated’ [6] where we began, for it is surely the outcome of the collaboration rather than the structure or process itself that counts.

**Figure 3.**
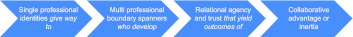
The conceptual framework.

This theoretical section has presented a limited review of just six concepts in collaborative working. These are; the One Children’s Workforce Tool, the importance of professional identities, the work of professionals in spanning boundaries and developing new forms of expertise, the importance of relational approaches in boundary spanning, the central position of trust in multi-professional working, and the success of these factors in creating collaborative advantage. These have created a conceptual framework that is shown in Figure 3.

## Methods

I had posed the questions, “to what extent are we working collaboratively for the benefit of children and young people?”, and secondarily for me, “to what extent can autoethnography be used to analyse collaborative practice?”. This research sought to understand the links between the conceptual framework outlined above and a real lived experience [27] of leadership. The data analysis would therefore need to establish to what extent the following concepts were evident in practice:

- Professional identities
- Boundary spanning
- Relational approaches
- Trust

and to what extent they led to collaborative advantage.

As the research is using personal experience as its unit of analysis it is interpretivist and post-positivistic, rejecting notions of a single truth. The “experiences” that I wished to analyse were encoded into a diary—a rich form of qualitative data. This is perhaps an example of the reflective writing that Winter et al. [28] advocate for professional learning. I strove for change and learning, and so framed it as practitioner action research [29] for myself (*via* my PhD) and the group I was working with. The research questions resonate with Kemmis’s [30] description of action research as a tool to reveal ‘architectures of practice’ as I examine the ‘saying, doings and relatings’ of my leadership of the multi-professional group, and the ‘sayings, doings and relatings’ of the group engaged in collaborative practice against the identified conceptual framework. Ellis and Bochner [31] refer to autoethnography as “*action research for the individual*”, and so I have framed my diary as an autoethnographic account that is subjective, emotive and full of researcher influence [31].

Ellis et al. [32] offer a definition of autoethnography as a systematic description and analysis of personal experience that allows understanding of wider cultural experiences, reflecting the aims of the practitioner action research I had embarked on. Autoethnography emphasises different ways of knowing from a post-modern tradition. Post modernism made it possible for critical theories to emerge and take hold in research and academia, and I like Wall:

*“find the relentless nudging of autoethnography against the world of traditional science holds wonderful, symbolic, emancipatory promise. It says what I know matters. How much more promise could it hold for people far more marginalised than I?”* [33].

Autoethnography can be seen as a form of narrative enquiry [34, 35] and it differs from narrative inquiry in that here, the researcher’s own experience is the only empirical data [36]. Bathmaker and Harnett [37] reveals how such personal stories can raise to the political, as:

*“possibilities for social change need, at least in part, to be understood and conceived of through the small everyday acts of individuals, and the histories that have brought them to their present place”* [37].

If my story does not reveal dominant discourses, it may at least connect with others experiences of leading multi-professional groups, just as Sparkes’s [38] autoethnographic account of chronic back injury resonated with other similarly injured individuals, validating their experience. Alaszewski states that diaries are:

*“the document par excellence, chronicling as it does the immediately contemporaneous flow of public and private events that are significant to the diarist”* [39].

And she is direct about them being fictional, creative works without audience in mind. The unique literary form of autoethnography means that past experiences are related biographically along with the study of the culture’s relational practices, helping insiders and outsiders to better understand that culture [39]. They are thick descriptions of culture. Sparkes [38] points out that because of the epistemological and ontological differences it makes no sense to judge texts from positivistic approach. Instead, Dumtrica [36] suggests that autoethnographers may rely on constructivist quality measures such as credibility, transferability, dependability and confirmability. This case study used such quality measures, and strove for empathetic validity. This is an excellent measure of quality of an interpretivist autoethnographic account where:

*“the traditional reverence for neutrality, objectivity and detachment may be out of place in forms of research that seek, or manage, to enhance human relationships”* [36].

As such, participant and peer validation was used to check whether it was credible (could you have experienced it?), whether it was valid (does it evoke feelings that it could be true and is it coherent?), and whether it was useful (is it helpful in any way?). I hoped that its publication would be a mark of it being generalisable (—does this specific illuminate general unfamiliar cultural practices?). Autoethnography has been criticised for being too artful, insufficiently rigorous, self-absorbed and narcissistic, but autoethnographers believe that research can be both rigorous, theoretical and emotional and inclusive of social and personal phenomena…and find it futile to debate whether it is a process or product, as the goal is to make the world we live in a better place [32]. The participant and peer validation process will go some way to answering this debate. Ethical clearance from the University of Cumbria was secured for the research and all difficulties associated with anonymity were also dealt with through the participant validation (and approval) of what was written.

The group that I was leading consisted of 20 professionals from seven organisations and sectors (education, health, social care, connexions, children’s services, the local safeguarding children’s board, the children’s trust, and the voluntary sector). The aim of the group was to ensure that the ‘children’s workforce’ in that local authority was able to meet the needs of children and young people. This was a massive and untenable brief from the start. The group met on a monthly basis for three hours, and all the professionals volunteered to come out of their home organisations to form this multi-professional team. Their organisations received no compensation for time away from regular responsibilities, nor were they allocated work time to carry out tasks from the workforce development group. This additionality was one barrier to effective working. Key tasks for the group were the analysis, strategic planning and implementation of workforce development tools and training that promoted integrated working. There were no additional resources for this, they had to be drawn into the workgroup by the members from their host organisations. This was a second barrier to effective working. I was a member of the team for a year before becoming a leader for 2.5 days a week over a six-month period. During this time I made entries to the diary on a weekly basis, reflecting back on key events that had occurred that week and were an average of 400 words long. It was a new methodological departure to use autoethnography in the context of interprofessional working.

Alaszewski [39] discusses two forms of diary analysis, a structural approach and a content approach. I first coded the autoethnographic diary with Kemmis’s [30] sayings, doings and relatings in order to make the architecture of practice visible. There were six codes developed here, a set of three codes related to what I said, did, and how I related to people as a leader, and a set of three related to what individuals said and did, and how they related to others in the group. I carried out a cross comparative analysis across the six categories to generate a first layer of findings. I then returned to the original data and carried out a second analysis, identifying and coding diary excerpts that related to the conceptual framework. These were then grouped and interpreted thematically. This two-stage analytical process revealed the architecture of practice (both espoused and real) in place and compared the practice to the concepts identified in the literature review.

## Results

### Stage one coding and analysis

The 6000 word text was coded into the six categories of leaders sayings, doings and relating, and the groups sayings, doings and there were many overlaps between the sayings and relatings, as the way that I related to people was verbal, and the ‘sayings’ column often ended up a repository for comments that did not fit in the other categories, but were things that I had ‘said’ in the diary. The categories of the Kemmis model were therefore not as straightforward to use for analysis as their simplicity suggested. Text was copied and pasted into the table below in a chronological order, and then grouped into common clusters of meaning. All categories are reported regardless of frequency. These are shown in Table 1 below.

### Stage two coding and analysis

The stage-two coding and analysis showed that there were numerous sayings, doings and relatings that could be linked to the six areas of the conceptual framework. These are summarised in Table 2 below, and then explored further and exemplified with full quotes in the discussion that follows.

## Discussion

### Stage one interpretation

Comparisons were then made between:

- The sayings, doings and relating of the leader
- The sayings, doings and relating of the group
- The sayings of the leader and the group
- The doings of the leader and the group
- The relating of the leader and the group

My sayings were based around empowering other people, and achieving things jointly, yet my doings were perpetuating individual rather than collaborative ways of working, I was ‘doing’ rather than ‘leading’, thus allowing other people to avoid tasks, and as a consequence I felt overwhelmed. Although valuing openness and honesty I was often complicit by not exposing other people’s criticisms. This duality between what I valued (integrity, inclusion, trust, collaborative advantage) and what I did led me to be very self-critical and to doubt my own legitimacy in the role. The complexity of the relationships led to personal and professional compromise as I attempted to keep the different members on board, causing me great frustration, and overshadowing the moments when I worked alongside others with similar values and working styles to great effect. Using Kemmis’s architecture of practice [30] therefore allowed me to see the differences between by conceptual knowledge and stated intentions and the reality of my daily practice. The analysis showed the difference between espoused and real practice effectively.

The analysis of the group comments showed that they engaged in lengthy debate that was pleasant and inclusive on the surface, but hid a layer of professional distrust and critique. These ‘sayings’ were evidence of poor relationships, characterised by a lack of trust. The outcome of this was evident in the behaviour of the group which was apathetic, avoidant and led to no action in some but not all cases, this clearly substantiates the conceptual framework, in that the lack of relational agency and trust led to collaborative inertia despite the experience of the group members acting as boundary spanners and multi professionals for a year.

Where I as a leader was critical of myself, the group were critical of each other (and I was sometimes complicit in that too by my lack of challenge to that behaviour). Where I overworked and had, arguably, too great a task focus, some of the group had none and did not engage in tasks. I worked well with those that were task focussed, open and honest. Despite my efforts to build alliances by smoothing water and taking the burden of tasks, some of the group only ‘played’ at getting on, and privately were critical of the group as a whole, and of individuals. This covert behaviour was divisive and characterised the lack of trust and relational agency above.

### Stage two interpretations

#### Professional identities

There is evidence that professional identities were a real challenge in this work. People were discounted and criticised and although these comments were not attached to other professions directly, there was a privileging of one professional discourse over another (although not evidenced in the diary), and hierarchies of power played out covertly in invitations to join certain group discussions outside of the official meeting forums. People did not seem to be discounted because of their sectoral professional background however—there were no comments about people being ‘social workers’ or ‘typical teacher!’ as often heard in early stage multi professional groups. The privileging and discounting revolved around how much influence the professionals had in their own organisations. As such, McKimm’s [7] model was not sufficiently sophisticated, a model of agency, or power enacted by boundary spanners was needed. It is interesting that the professionals used this new role description as a discriminator, and where they perceived colleagues to be lacking in skills, legitimacy or authority, it led to division:

“X has just dragged me out to lunch for yet another rant about the inability of C to make change happen in their own organisation. I am tired of such bickering and of sneaky conversations.”

My own professional identity as a leader was evidently weak, and repeated difficulties led me to question my own capabilities:

“I was worried at my ability to hold them together into a single group, my authenticity and legitimacy as their leader”.

This perhaps does reflect something of the difficulties that the literature suggested multi professionals may experience in new situations, or it could be a reflection of my anticipation that accusations that they cast at others may be levelled against me. There was however no open challenge to my role as leader at any point.

### Boundary spanning

Arguably the members of this group needed to be able to lead within their own and across other organisations. The inability of some members to do this led to criticism and discounting as mentioned above. ‘Discounting’ is a term that refers to the psychological process of ignoring the relevance or importance of an individual or their ideas [40]. It was uncertain whether they were not able to lead due to a lack of power, a lack of time, or a lack of motivation to effect change. This created the most tension. This led to various members of the group questioning the legitimacy of the membership of the group, again covertly, as they did not seem able to make decisions, influence or make changes in their organisations. It seems then that either the organisations were too ‘bonded’ or the individuals did not have the necessary complex skill set. In my diary even I raise the issue:

“Organisational boundaries are slowing us down! People are not able to get permission to do stuff—even to agree to support championing children in their own organisations. They don’t want to ask awkward questions about induction processes, are worried about promoting courses—I mean—these are supposed to be champions!!!!”, and;

“If workforce leads are facing differences in their own organizations then the whole thing will stall…the nub of the issue seems to be how much authority and legitimacy the members have back in their own organisations.”

The evidence does not lead to a conclusion either way. The workforce group itself was ‘bridged’ as it had a clear outward focus [11], but despite rich and lengthy dialogue and shared artefacts [9] such as the One Workforce Tool, some members of the group did not reach into their own organisations. Perhaps as Noble and Jones suggested their host organisations have different cultures, levels of autonomy and attitudes to risk [10], perhaps they were too bonded, or perhaps the boundary spanning skill set as outlined in the theoretical review were too demanding as some individuals did not seem to enact the skills [15] that they had within their own and other organisations. I suggest that these professionals did have the skill set, and some had greater or lesser organisational boundaries to overcome, what was critical therefore was not just the skill set, but also the power, or ‘agency’ to enable them to take actions.

### Relational approaches to collaboration

Taking a relational perspective seems to explain the difficulties of the group. Although the relevant skills and experiences were in place, the outward focus of the group, and the ‘hybrid’ nature of the members, there was a lack of functioning. This is exemplified by this statement in my diary:

“Members seemed to pick up on the political atmosphere and were negative, non-committal and indecisive. It was SO frustrating. As if the more they are shown what they can do, the less they then want to do it….why such apathy after such a great event last week?”

This team has the ‘know what’, ‘know how’ and ‘know who’ that was shown to comprise ‘relational agency’ by Edwards [14], but they are hampered by interpersonal issues. The result of this is that the ‘know how’ pertains only to surface behaviours that mask deep biases and interpersonal issues. This theme was the most prevalent in the autoethnographic account. Some of the interpersonal issues were caused by a covert conversation—these show weak interpersonal skills and a lack of openness and trust. On the one hand; “*Past issues arose and everyone agreed openly to stop referring to past issue and to move on*.”, yet there remained “*a range of mixed messages*”. I was frequently drawn into trying to diffuse these issues in a ‘mediating’ role. This was often because people held beliefs about one another that they did not share openly. Here are some examples:

“A sent out an agenda for the next meeting—I encouraged her to do so to start to step up as the successor. What a disaster. B hit the roof about how shabby it was—to me not to A. What a nightmare.”

“C has sent some really ‘off’ emails to both me and D about things that he disagrees with…….he’s entitled to his opinions, but there are ways of saying that you disagree! His views on the event risk the whole enterprise as D is taking it as a direct attack and evidence that he does not believe that she is competent.”

“They both seem to distrust one another, jump to the worst possible conclusion every time and believe that the other one hates them.”

These disagreements had a profound impact on the success of the group as they got in the way of effective multi professional working—they would have got in the way of any group working, multi professional or not. Poor interpersonal relationships were exhibited in covert conversations, and a break-down of open and honest dialogue, reinforcing Reder and Duncan’s [19] views about the centrality of communication skills in Child Abuse Reviews. As a leader I could have exposed the situation and triggered an open dialogue about the difficulties, but instead I also perpetuated the culture that annoyed me so much. At times this was because of the difficulty of managing volunteers:

“I have realised that I dislike asking people to do things—it’s complicated leading people who are basically volunteers—normal authority and responsibility does not work, a subtle type of value laden leadership is needed to appeal to them to help out. Its slow and frustrating and I see the same people doing tasks and the same people avoiding them.”

But that was not always the case, sometimes I just did not want to offend people: “*Really peed off, but of course didn’t say so!!!”.* I was aware and confused by the appropriateness of my lack of honesty at times:

“I was however aware that I down played my conversation with P so that I didn’t have to admit that we had been talking about changing a communication that LO sent out….coward? liar? Diplomat?”.

Perhaps then, whilst I declare myself to be an honest and open leader, I did engage in the ‘mobilisation’ of the team, and also the ‘politicking’ and ‘manipulation’ that Huxham and Vangen [21] described in collaborative leadership situations. I questioned my own actions as a mediator:

“I think it shows that I build rapport and empathy with everyone and then try to move them into a more helpful space where they understand one another…its peacekeeping rather than two faced…may be I should stop rescuing and let them fight it out?”, and constantly worried as to whether this was appropriate leadership, or ducking the issue.

### Trust

Trust can be seen as the central issue in all the interpersonal issues. If the group really trusted one another then they would be able to discuss the issues that they were having together, openly. Managing the interpersonal issues was exhausting and arguably took up the majority of my energy if not time in this project. Much more could have been achieved if the team were open, honest, trusting and got on with one another. Publically trust was incentive driven (e.g., by trusting one another we can get more done), but covert conversations revealed that really the trust was ‘avoiding’ being viewed as negative and some members of the team really did not trust others unless it would be harmful to be seen as withholding it. This shows the value of Perri et al. [24]. Use of Huxham’s [23] trust matrix may have enabled me to see ways to build quick wins and move the team forward. It was a lack of trust that meant that the professionals criticised one another’s membership as they did not trust them to effect change back in their own organisations. A lack of trust was exhibited in and fuelled by interpersonal issues. Covert conversations created an undercurrent that others could detect. And the lack of trust in the group led me to continually question my own leadership as I started to lack trust in my own ability. This can be shown as a powerfully negative trust spiral, each poor experience diminishing future trust, an inverse of Huxham’s [23] iterative trust building model.

### Collaborative advantage

Needless to say after the discussion above, collaborative advantage was not achieved. My last diary entry was:

“Final WD meeting was a complete nightmare—worst meeting ever. I can hardly bear to write about it, what an end!”

The professionals had all moved from being single professionals to being multi professionals, but a range of boundary spanning skills, and organisational boundaries made work difficult for some, and the lack of relational agency and trust across the entire group prevented collaborative advantage from being achieved. This shows the relevance of the conceptual framework in Figure 3. Huxham and Vangen [21] wrote about the importance of managing the ambiguity, dynamics and membership of collaborative groups with a full range of leadership styles in order to achieve collaborative advantage. In this situation, the group seems to have managed the ambiguity of the context themselves, and they seemed to be coping with the dynamic nature of change, so this was not the key barrier to collaborative advantage. Membership for this group was key as individuals discounted the ability of others to work within their own organisations and to boundary span. These differences were then dealt with covertly due to a lack of trust in the group. There are two implications of this—either the membership needed to be refreshed to include people with boundary spanning skills that had relational agency and could build trust, or the current group members needed to be open to change. The group had many very successful outputs, but these tended to be driven and achieved by individuals who wanted to make a difference that had the most drive, and the most relational agency, working in small groups or pairs. These tasks then were carried out more in partnership than in collaboration. The group as a whole did not achieve collaborative advantage, but collaborative inertia, with lengthy meetings and discussions having negligible joint outputs. Perhaps painfully, I need to reflect on whether I contributed to these negative working styles by missing opportunities to tackle the issues head on.

Presenting the findings of this research to the group was challenging, and presented me with the very opportunity that I had previously overlooked or avoided. The fact that I had been a participant in the research (rather than doing research ‘on’ them) made the findings easier to bear, as there were an equal number of criticisms of myself as of them. Presenting the research autoethnographically also meant that I could own the research as a biased account, and so easily invite them into a critical discussion of their experiences and perceptions. I did not present this as ‘what is’, but ‘what might be from my perspective’. There are a number of actions that arise from this research. Primarily for the group, there was an open and frank discussion about the difficulties of working together that previously went unnamed, and a commitment to address this and work towards collaborative advantage through further developmental, participatory action research. For myself there was great learning about my attributes and short falls as a leader—it highlighted how easily one can unwittingly collude with the status quo of any situation and with policy. When I carried out this research I truly realised and benefitted from the critical consciousness that is intrinsic to research endeavours. I saw how useful and how limited by conceptual framework had been and saw the reality of the situation I had led and of the policy context with greater clarity. This research has also led me to realise the importance of human agency within collaborative settings, and has opened up a whole new line of enquiry within my PhD.

## Conclusions

Using Kemmis’s [30] framework of the architecture of practice made practice observable. The groups public sayings were of principled work and commitment to collaboration, but the private sayings were covert and complicit and did not reflect the principles that the group, and certainly I aspired to. The doings were therefore clouded with interpersonal issues and inertia as people were not truly engaged, did not truly trust one another and did not feel able to contribute. The relatings prevented effective doings. This bolsters the evidence across the interprofessional field of the importance of communication and relational agency, as without it, even the most experienced, committed and dedicated multi professionals may flounder. The conceptual framework that was identified through a review of theory needs further development, yet even at this early stage, it was a useful analytical frame showing both the shortfalls of national policy and the reasons why the group were not attaining collaborative advantage. Notions of boundary spanning are extended by the concept that they need not only a hybrid experience, and unique skill set, but that boundary spanning individuals also need a source of legitimate power in their own and other organisations, and a sense of ‘agency’. This opens up a new line of enquiry. The action research led to personal change as there were key realisations for me about the way in which I lead multi professional groups, and new understandings to shape my PhD work on collaboration. There was also change for the group, as my presentation of the findings initiated a new participative action research project within the group. The research endeavour had not only evidenced the extent to which the group achieved collaborative advantage, but also generated insights into why it was not being achieved. Although these are perhaps not new findings for the field of interprofessional work, the use of autoethnography to document an interprofessional working was new. The process allowed naturalistic research to take place. The process of keeping an autoethnographic diary was very achievable as a participant researcher. Analysis of the diary with the two-part analysis showed how the autoethnography did capture key insights into the groups working. Perhaps most significantly was that the presentation of the findings from a personal account was very palatable to the group and led to dialogue, debate, critical consciousness and change. I invite further professionals working in integrated settings to capture what it is that they do through autoethnographic accounts, developing the methodology and its applications further.

## Figures and Tables

**Table 1.** Summary of themes that emerged from the first stage coding.

	Sayings	Doings	Relating
**Leader**	Language of influence used	Multiple attempts at single communication	Broker between individuals who disagree
	Personal legitimacy questioned	Overwhelmed	1:1 relationships
	Self critique	Did tasks that others should do	Seek reassurance
Overwhelmed	Rescue others through personal task orientation	Enjoy working with people similar to me
	Empower, value and celebrate others	Small meetings and 1:1 communication	Importance of trust
	Dishonest by silence	Complicit by not airing grievances	Frustration at others criticisms of one another, unable to surface it so complicit
	Lots of TRYING to manage process	Unable to delegate to volunteers
**Group**	Mixed messaged leading to confusion	Avoidance	Easy relationships when all agree
	Contradictions in a short space of time	No power in organisations so no changes	Covert criticisms, face to face pleasantries
	Distancing from tasks or one another	Slow progress	Difficult email communications
	Covert criticisms	Apathy	Lack of trust
	Lengthy inclusive debates		

**Table 2.** 

	Sayings	Doings	Relatings
Single professional identities	Publically supportive Privately discounting	Covert behaviour that was unsupportive of others Privileged discourses from some professionals	Superficial—personally fine, professionally issues. Hierarchical
Multiple, boundary spanning professionals	Criticised if not able to ‘bridge’ or boundary span Questioned other people’s rights to be members Raised the lack of legitimacy or power that individuals have	People not working across organisational boundaries Used the One Workforce Tool to mediate new expertise	Duplicitous Political
Relationships, communication and trust	Espoused the values of trust and openess	Lack of skill Lack of relational agency Did not enact trust and openness	Lacked authenticity Surface level communications
Collaborative advantage	Awareness of the concept and how to achieve it	Unable to enact as blocked by lack of trust, legitimacy and boundary spanning skills	Inert relationships
